# New Instrument, by Prof. John T. Hodgin

**Published:** 1869-02

**Authors:** 


NEW INSTRUMENT.—Prof. John T. Hodgins, of St Louis, has invented an instrument which he calls a *Snow*, for the removal of foreign bodies from the urethea. We have not seen the instrument, but from his description of it we have no doubt but that it will answer the purpose for which it is intended.

